# Spontaneous immunological targeting of endogenous retrovirus K is directed against the envelope protein in rhesus macaques

**DOI:** 10.1186/1742-4690-8-S2-P60

**Published:** 2011-10-03

**Authors:** Enrique J Léon, Lyle T Wallace, Francesca A Norante, Matthew B Buechler, Laura P Newman, Philip A Castrovinci, Brad R Jones, Robert J Gifford, Jonah B Sacha

**Affiliations:** 1 Vaccine and Gene Therapy Institute, Oregon Health & Science University, Beaverton,OR, USA; 2 Oregon National Primate Research Center, Oregon Health & Science University, Beaverton, OR, USA; 3 Departmentof Pathologyand Laboratory Medicine, University of Wisconsin - Madison, WI, USA; 4 Department of Immunology, University of Toronto, Toronto, ON, Canada; 5 Aaron Diamond AIDS Research Center, Rockefeller University, New York, NY, USA

## 

Endogenous retroviruses (ERVs), remnants of ancient retroviral infections, have invaded the germ line of every vertebrate species including both non-human primates and humans. Most ERVs are functionally crippled by extensive deletions, mutations and hypermethylation, leading to the view that they are inert genomic fossils. However, some ERVs retain the potential for biological activity and can produce mRNA transcripts, functional viral proteins, and even non-infectious virus particles during certain developmental and pathological processes. Although ERV gene products are implicated in the etiology of disease, their impact during host infection with exogenous retroviruses such as human and simian immunodeficiency virus (HIV/SIV) remains unknown. Here, we identify the homologue of the biologically active human ERV-K, macaque ERV-K(macERV-K), which retainsintact open reading frames in the macaque genome. Indeed, we isolated a fully-spliced mRNA product encoding macERV-K Env, which contains all the structural features of a canonical retroviral Envelope protein. Furthermore, we identified robust CD8+ and CD4+ T cell responses directed at macERV-K Env in one SIV-infected Indian rhesus macaque. The macERV-K Env-specific CD4+ T cell response in this animal was larger than the entire 5lV-specific CD4+ T cell response and had a uniform effector memory phenotype, indicating continual low-level antigen exposure. In support of this, we also identified antibody responses targeting macERV-K in this animal. Although SIV infection did not appear to trigger ERV Env-specific T cell responses, these data suggest that Env proteins from ERVs can be active during infections with exogenous retroviruses. The interaction of ERV Env proteins expressed in HIV-infected cells may have major implications for the pathogenicity of HIV and warrants further investigation.

